# Pharmacological polysulfide suppresses glucose-stimulated insulin secretion in an ATP-sensitive potassium channel-dependent manner

**DOI:** 10.1038/s41598-019-55848-7

**Published:** 2019-12-18

**Authors:** Tomohiro Shoji, Mikio Hayashi, Chisato Sumi, Munenori Kusunoki, Takeo Uba, Yoshiyuki Matsuo, Hideo Kimura, Kiichi Hirota

**Affiliations:** 1 Department of Anesthesiology, Kansai Medical University, Hirakata, Japan; 2 Department of Human Stress Response Science, Institute of Biomedical Science, Kansai Medical University, Hirakata, Japan; 3 Department of Cell Physiology, Institute of Biomedical Science, Kansai Medical University, Hirakata, Japan; 4 Department of Pharmacology, Faculty of Pharmaceutical Science, Sanyo-Onoda City University, Sanyo-Onoda, Japan; 5 Department of Molecular Pharmacology, National Institute of Neuroscience, National Center of Neurology and Psychiatry, Kodaira, Japan

**Keywords:** Mechanisms of disease, Diabetes complications

## Abstract

Hydrogen sulfide (H_2_S) is an endogenous gaseous transmitter synthesized in various cell types. It is well established that H_2_S functions in many physiological processes, including the relaxation of vascular smooth muscle, mediation of neurotransmission, regulation of inflammation, and modulation of insulin signaling. In recent years, it has been revealed that polysulfides, substances with a varying number of sulfur atoms (H_2_Sn), are generated endogenously from H_2_S in the presence of oxygen. A series of studies describes that sulfane sulfur has the unique ability to bind reversibly to other sulfur atoms to form hydropersulfides and polysulfides, and that polysulfides activate ion channels and promote calcium influx. Furthermore, polysulfides regulate tumor suppressor activity, promote the activation of transcription factors targeting antioxidant genes and regulate blood pressure by vascular smooth muscle relaxation. Insulin secretion from pancreatic β cells plays a critical role in response to increased blood glucose concentration. H_2_S has emerged as an important regulator of glycemic control and exhibits characteristic regulation of glucose homeostasis. However, the effects of polysulfides on glucose-stimulated insulin secretion (GSIS) are largely unknown. In this study, we demonstrated that pharmacological polysulfide salts including Na_2_S_2_, Na_2_S_3_, and Na_2_S_4_ considerably inhibit GSIS in mouse and rat pancreatic β-cell-derived MIN6 and INS-1 cell lines, and that the effect is dependent on the activation of ATP-sensitive potassium channels. In addition, we demonstrated that a mixture of Na_2_S and diethylamine NONOate inhibits GSIS in a similar way to the pharmacological administration of polysulfide salts.

## Introduction

Hydrogen sulfide (H_2_S) is synthesized endogenously in various cell types, and functions as an endogenous gaseous transmitter. H_2_S function is well established in many physiological processes, including the relaxation of vascular smooth muscle, mediation of neurotransmission, regulation of inflammation, and modulation of insulin signaling^1–3^. In recent years, it has been revealed that polysulfides, substances with a varying number of sulfur atoms (H_2_S_n_), are generated from H_2_S in the presence of oxygen^1,4,5^ and by 3-mercaptopyruvate sulfurtransferase (3MTS)^6^. In addition, polysulfides are also generated by the interaction between H_2_S and nitric oxide (NO)^7^. Polysulfides contain sulfane sulfur, which is present in various proteins and functions as a potential intracellular H_2_S store that releases H_2_S under reducing conditions. A series of studies describe the unique ability of sulfane sulfur to bind reversibly to other sulfur atoms to form hydropersulfides (R-S-SH) and polysulfides (-S-S_n_-S-)^1^. Polysulfides have been shown to activate ion channels and promote calcium influx, as well as to regulate tumor suppressor activity, promote the activation of transcription factors targeting antioxidant genes, and modulate blood pressure by vascular smooth muscle relaxation^8^. H_2_S and sulfane sulfur constitutively coexist, and recent work suggests that sulfane sulfur species may act as signaling molecules in at least some biological processes^9^. It is also clear that many of the effects of H_2_S are mediated through reactions with cysteine sulfur on regulatory proteins and most of these are not mediated directly by H_2_S but require prior oxidation of H_2_S and the formation of H_2_S_n_^10^.

H_2_S has also emerged as an important molecule in glycemic control, exhibiting characteristic regulation of glucose homeostasis. Specifically, H_2_S stimulates glucose production via activation of gluconeogenesis and glycogenolysis in hepatocytes, yet inhibits lipolysis in adipocytes and insulin secretion from pancreatic β cells^11–13^. Currently, however, the effects of polysulfides on glucose-stimulated insulin secretion (GSIS) are largely unknown.

In this study, we investigated the effect of pharmacological polysulfide salts including sodium disulfide (Na_2_S_2_), sodium trisulfide (Na_2_S_3_), and sodium tetrasulfide (Na_2_S_4_) on insulin secretion under low and high glucose conditions using cell biological and electrophysiological methods. We examined the effects of polysulfides at basal conditions and during GSIS and demonstrated that polysulfides activate ATP-sensitive potassium (K_ATP_) channels, suppressing GSIS in mouse insulinoma 6 (MIN6) cells, rat insulinoma 1 (INS-1) cells, and mouse pancreatic β-cells/islets.

## Results

### Polysulfide salts inhibit glucose-stimulated insulin secretion in MIN6 cells

First, insulin secretion in response to changes in extracellular glucose concentration was examined in MIN6 cells. Cells were pre-cultured under 2 mM glucose conditions for 0.5 h. Then, the cells were exposed to a range of glucose concentrations (5, 10, and 20 mM) for 1 h, and the concentration of insulin in the culture media was assayed (Supplementary Fig. 1a). Next, the time-dependent GSIS profile was investigated. To do this, cells were exposed to 20 mM glucose and insulin concentration was assayed at 0, 20, 40, 60, and 120 min (Supplementary Fig. 1b). Glucose-stimulated insulin secretion was clearly observed in MIN6 cells.

**Figure 1:**
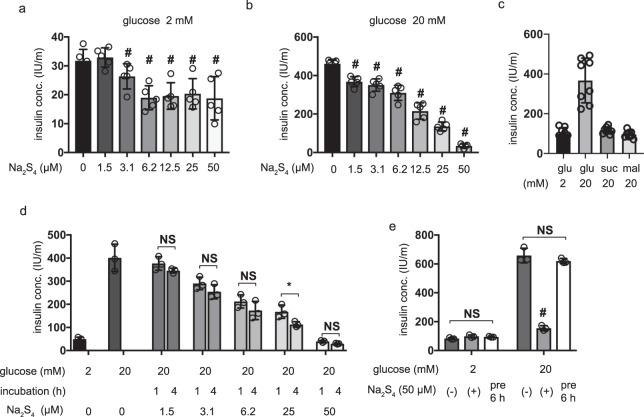
Dose- and time-dependent effects of polysulfide salt, sodium tetrasulfide (Na_2_S_4_) on glucose-stimulated insulin secretion in MIN6 cells. (**a,b**) Mouse MIN6 cells were exposed to Na_2_S_4_ (0, 1.5, 3.1, 6.2, 12.5, 25 and 50 µM) for 4 h under 2 mM glucose conditions, and insulin secretion was determined in the presence of 2 mM (**a**) or 20 mM glucose (**b**) (n = 5). (**c**) MIN6 cells were treated with 20 mM glucose (glu), sucrose (suc) or maltose (mal) for 1 h (n = 8). (**d**) MIN6 cells were exposed to Na_2_S_4_ (0, 1.5, 3.1, 6.2, 25, and 50 µM) for 1 h or 4 h and then insulin secretion was determined under 20 mM glucose conditions. (n = 5) (**e**) MIN6 cells were exposed to Na_2_S_4_ for 4 h and then to 20 mM glucose or incubated for 6 h without Na_2_S_4_ and then insulin secretion was determined in 20 mM glucose conditions (n = 3). Data are presented as mean ± SD. Differences between treatments were evaluated by one-way ANOVA followed by Dunnett’s test for multiple comparisons; ^#^*P* < 0.05, as compared with the control: (Na_2_S_4_ 0 µM: a and b). *P < 0.05 for comparison of the indicated groups (c, d, and e).

Next, the effect of the polysulfide salt Na_2_S_4_ on GSIS was investigated. MIN6 cells were exposed to 1.5–50 µM Na_2_S_4_ for 4 h under 2 mM glucose conditions and then the insulin concentration in the culture media was assayed. Na_2_S_4_ at concentrations exceeding 3.1 µM significantly inhibited basal insulin secretion in MIN6 cells under 2 mM glucose conditions (Fig. 1a). MIN6 cells were then exposed to Na_2_S_4_ for 4 h under 2 mM glucose conditions and then 20 mM glucose conditions for 1 h. Here, Na_2_S_4_ concentrations exceeding 1.5 µM significantly inhibited GSIS in MIN6 cells (Fig. 1b). The insulin secreted in response to the polysaccharides sucrose and maltose was also assessed. Only glucose elicited insulin secretion, indicating that other polysaccharides do not largely impact the secretion of insulin (Fig. 1c). Subsequently, the effect of changing the Na_2_S_4_ incubation time was investigated. MIN6 cells were preincubated with Na_2_S_4_ for 1 h or 4 h and GSIS was measured. No significant differences were detected, but only under 25 µM (Fig. 1d). Finally, the reversibility of the inhibitory effect of Na_2_S_4_ was investigated. MIN6 cells were either exposed to Na_2_S_4_ for 4 h and then to 20 mM glucose, or incubated for 6 h without Na_2_S_4_ and then exposed to 20 mM glucose. The inhibitory effect of Na_2_S_4_ was completely recovered after 6 h of incubation (Fig. 1e).

**Figure 2:**
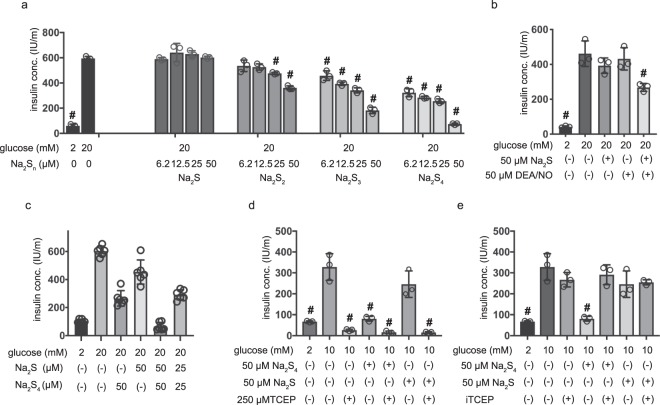
Effects of hydrogen sulfide donor and polysulfide salts on glucose-stimulated insulin secretion in MIN6 cells and its dependency of S-S bonds. (**a**) Mouse MIN6 cells were exposed to Na_2_S, Na_2_S_2_, or Na_2_S_3_ (0, 6.2,12.5, 25 and 50 µM) for 1 h in the presence of 2 mM glucose and then insulin secretion was determined in 20 mM glucose. (n = 3) (**b**) MIN6 cells were exposed to Na_2_S or DEA/NO in 2 mM or 20 mM glucose. (n = 3) (**c**) MIN6 cells were exposed to Na_2_S or Na_2_S_4_ in 20 mM glucose. (n = 3) (**d,e**) MIN6 cells were exposed to 50 µM Na_2_S or 50 µM Na_2_S_4_ with 250 µM tris (2-carboxyethyl) phosphine hydrochloride (TCEP) (**d**) or Na_2_S or Na_2_S_4_ treated by immobilized-TCEP (iTCEP). (n = 3) (**e**) for 1 hour and then insulin secretion was determined under 20 mM glucose conditions. (n = 3) Data are presented as mean ± SD (n = 3). Differences between treatments were evaluated by one-way ANOVA followed by Dunnett’s test for multiple comparisons; ^#^*P* < 0.05, as compared with the control (0 µM Na_2_S_4_: 20 mM or 10 mM glucose)

The effect of other sulfane sulfur donors on GSIS, including Na_2_S_2_ and Na_2_S_3,_ was also examined in MIN6 cells. Na_2_S_2_ or Na_2_S_3_ both inhibited GSIS within 4 h of their addition to the media (Fig. 2a). Remarkably, the inhibitory effect of Na_2_S_2_ (IC50 > 250 µM) was weaker than that of Na_2_S_3_ (IC50 = 39.9 µM) and the effect of Na_2_S_3_ was weaker than that of Na_2_S_4_ (IC50 = 9.5 µM). Finally, the effect of H_2_S was investigated. In contrast to sulfane sulfur donors, Na_2_S did not significantly affect GSIS at any concentration up to 50 µM (Fig. 2a).

### The inhibition of GSIS by polysulfides generated by the interaction of H_2_S with NO

The oxidation of H_2_S generates H_2_Sn, and the interaction of H_2_S with S-nitroso cysteine generates cysteine persulfide. In fact, the interaction of H_2_S with NO directly produces H_2_S_n_^7^. Here, MIN6 cells were exposed to a mixture of Na_2_S, a sodium salt of sulfide, and diethylamine NONOate (DEA/NO), a donor of NO that generates polysulfides. Neither 50 µM Na_2_S nor 50 µM DEA/NO inhibited GSIS in MIN6 cells (Fig. 2b). On the other hand, a mixture of 50 µM Na_2_S and 50 µM DEA/NO significantly inhibited GSIS, as did 12.5 µM Na_2_S_4_ (Fig. 2b). We investigated the synergistic role of polysulfides (Na_2_S_4_) and H_2_S (Na_2_S) on GSIS, and determined that the combination of Na_2_S_4_ and Na_2_S resulted in an additive effect (Fig. 2c).

### Pretreatment with reducing agent abolishes the inhibitory effect of Na_2_S_4_

In this study, we have shown that the potency of inhibition is correlated with the number of disulfide bonds, the effect of a reducing agent (tris (2-carboxyethyl) phosphine hydrochloride (TCEP)) that cleaves disulfide bonds was assessed. First, TCEP was added directly into the culture media, and measured insulin levels remained very low (Fig. 2d). However, we also found that TCEP abolishes the immunogenicity of insulin by cleaving the S-S bond in insulin (Supplementary Fig. 2). Therefore, Na_2_S_4_ was pretreated with immobilized-TCEP (iTCEP) and then administered to cells. The pretreated Na_2_S_4_ did not affect the immunogenicity of insulin (Supplementary Fig. 2) but displayed reduced inhibitory potency in terms of GSIS (Fig. 2e). These results indicate that disulfide bonds in polysulfides are essential for their inhibitory effects on GSIS.

### Polysulfide salts inhibit GSIS in INS-1 cells and mouse pancreatic β-cells/islets

**Figure 3:**
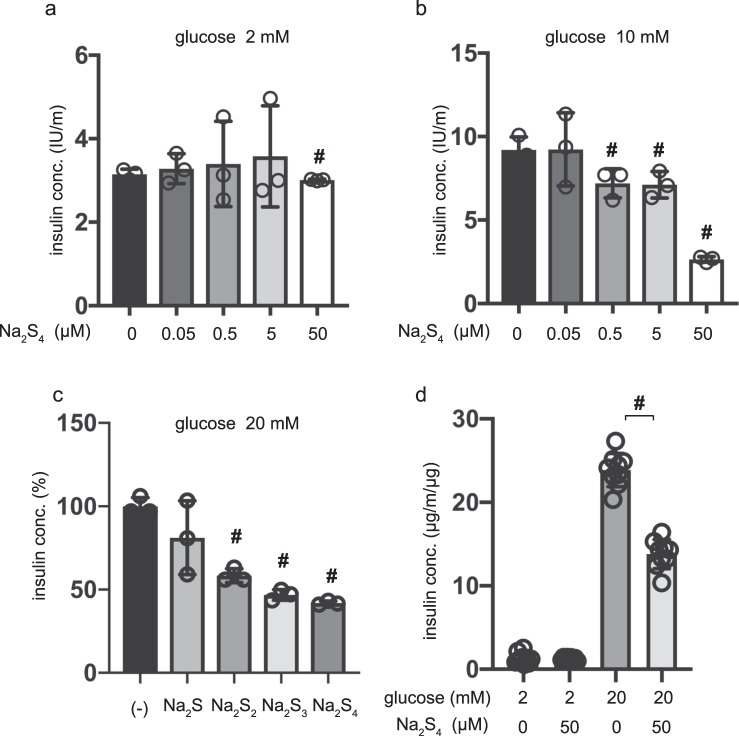
Dose- and time-dependent effects of polysulfide salts on glucose-stimulated insulin secretion in INS-1 cells and mouse pancreatic β-cells/islets. (**a,b**) Rat insulinoma INS-1 cells were exposed to Na_2_S_4_ (0, 0.05, 0.5, 5, or 50 µM) for 1 h in the presence of 2 mM glucose and then insulin secretion was determined under 2 mM (**a**) or 10 mM (**b**) glucose conditions. (n = 3) (**c**) INS-1 cells were exposed to 50 µM Na_2_S, Na_2_S_2_, Na_2_S_3_, or Na_2_S_4_ for 1 h in 2 mM glucose and then insulin secretion was determined under 20 mM glucose conditions (n = 3). (**d**) Mouse pancreatic β-cells/islets were exposed to 50 µM Na_2_S_4_ for 1 h in 2 mM or 20 mM glucose (n = 3). In the case of β-cells/islets insulin concentrations were compensated with total protein weight. Data are presented as mean ± SD (n = 3). Differences between treatments were evaluated by one-way ANOVA followed by Dunnett’s test for multiple comparisons; ^#^*P* < 0.05, as compared with the control (Na_2_S_3_ or Na_2_S_4_ 0 µM).

To investigate whether the suppressive effect of polysulfides can be observed in other cell types, we used the rat insulinoma INS-1 cell line and mouse β-cells/islets. The effect of Na_2_S_4_ on GSIS was investigated in INS-1 cells. As in the case of MIN6 cells, Na_2_S_4_ inhibited GSIS in a dose-dependent manner (Fig. 3a,b). Na_2_S_2_ and Na_2_S_3_ also inhibited GSIS (Fig. 3c). β-cells/islets were incubated with 50 µM of Na_2_S_4_ in 2 mM glucose from 30 min to 1 h, and the insulin concentration was assessed. The insulin concentration of β-cells/islets, were compensated with total protein weight. As with MIN6 and INS-1 cells, 50 µM Na_2_S_4_ significantly suppressed IS after incubation for 4 h (Fig. 3d). When cells were exposed to 20 mM glucose, 50 µM Na_2_S_4_ significantly suppressed GSIS after 1 h of incubation. Thus, the inhibitory effect of polysulfides on GSIS is not only observed in mouse MIN6 cells, but also in rat INS-1 cells and β-cells/islets, suggesting that this phenomenon may be universal.

### Effect of polysulfides on death of MIN6 cells

**Figure 4:**
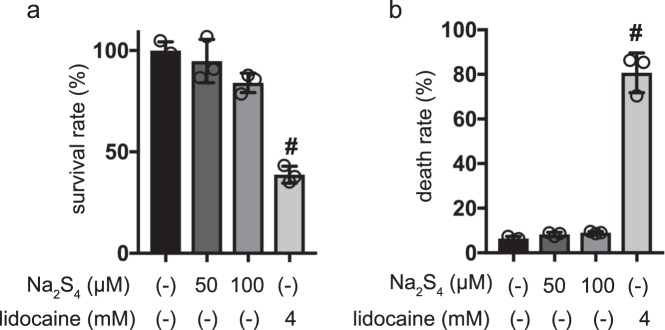
Impact of Na_2_S_4_ on death of mouse MIN6 cells. (**a,b**) MIN6 cells were exposed to Na_2_S_4_ at doses from 0 µM to 100 µM and to lidocaine as positive control at 10 mM and cultured for periods ranging from 0 h to 4 h prior to cell viability evaluation. Cell death was also assayed by flow cytometry (**a**) and trypan blue exclusion method (**b**). Differences between treatments were evaluated by one-way ANOVA followed by Dunnett’s test for multiple comparisons; ^#^*P* < 0.05, as compared to the control cell population (Na_2_S_4 ;_0 µM, lidocaine; 0 mM treatment).

Cell death was assayed by flow cytometry and the trypan blue exclusion method. 4 mM lidocaine treatment induced cell death, as detected by propidium iodide (PI) or Annexin V staining. However, neither 50 µM nor 100 µM Na_2_S_4_ induced cell death within 1 h (Fig. 4a). In the case of the flow cytometry assessment method, neither 50 µM nor 100 µM Na_2_S_4_ induced cell death within 1 h, whereas lidocaine induced widespread cell death (Fig. 4b, Supplementary Fig. 3).

Our findings show that polysulfides at 50 µM did not cause MIN6 cell death and indicate that the observed suppression of GSIS is not due to cell toxicity caused by polysulfides.

### Effects of polysulfide salts on cellular energy metabolism in MIN6 cells

**Figure 5:**
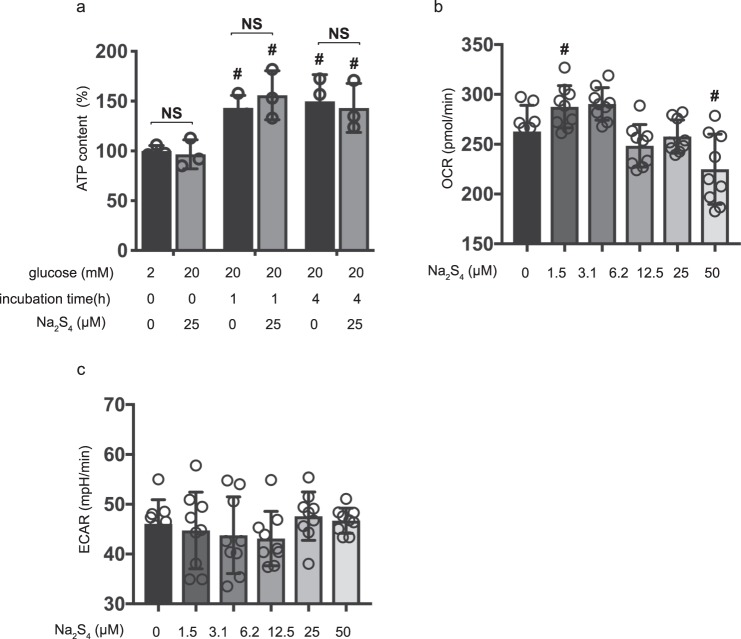
Effects of Na_2_S_4_ on cellular energy metabolism. (**a**) Mouse MIN6 cells were cultured from 1 h to 4 h with 25 µM Na_2_S_4_ prior to determination of the cellular ATP level (n = 3) under 2 mM or 20 mM glucose conditions. (**b,c**) Mouse MIN6 cells were exposed to Na_2_S_4_ (0, 1.5, 3.1, 6.2, 12.5, 25 and 50 µM) for a period of 4 h, followed by oxygen consumption rate (OCR) assay (**b**) and extracellular acidification rate (ECAR) assay (**c**) (n = 9). Differences between treatments were evaluated by one-way ANOVA followed by Dunnett’s test for multiple comparisons; ^#^*P* < 0.05, as compared to the control cell population (Na_2_S_4_ 0 µM, glucose 20 mM). *P < 0.05 for comparison of the indicated groups.

Intracellular ATP concentration plays a crucial role in GSIS. High extracellular glucose increases ATP concentration in pancreatic β-cells^14,15^. Here, in agreement with previous studies, we observed an Na_2_S_4_ dependent effect on intracellular ATP concentaration upon exposure to 2 mM or 20 mM glucose at the timepoints 1 and 4 h. 20 mM glucose increased ATP concentration compared to 2 mM glucose. However, 25 µM Na_2_S_4_ did not affect ATP concentration at all (at all timepoints) in cells treated with 20 mM glucose (Fig. 5a).

It has been reported that glucose increases oxygen consumption in mitochondria and stimulates oxidative phosphorylation (OXPHOS) in mitochondria^16,17^. We therefore investigated the effect of Na_2_S_4_ on oxygen metabolism in MIN6 cells (Supplementary Fig. 4a). High-glucose stimulation activated mitochondrial respiration in MIN6 cells (Supplementary Fig. 4b). The oxygen consumption rate (OCR) was increased in 20 mM glucose conditions (Supplementary Fig. 4b). The extracellular acidification rate (ECAR) also increased in 20 mM glucose conditions (Supplementary Fig. 4c). A concentration of 1.5–25 µM Na_2_S_4_ did not affect OCR or ECAR under 20 mM glucose conditions within the 4 h measurement period (Fig. 5b,c). However, 50 µM Na_2_S_4_ suppressed OCR in response to exposure to high glucose. Together, these results show that polysulfides applied to cells at concentrations up to 25 µM did not affect cellular energy or oxygen metabolism.

### Effects of polysulfides on insulin secretion induced by glibenclamide

**Figure 6:**
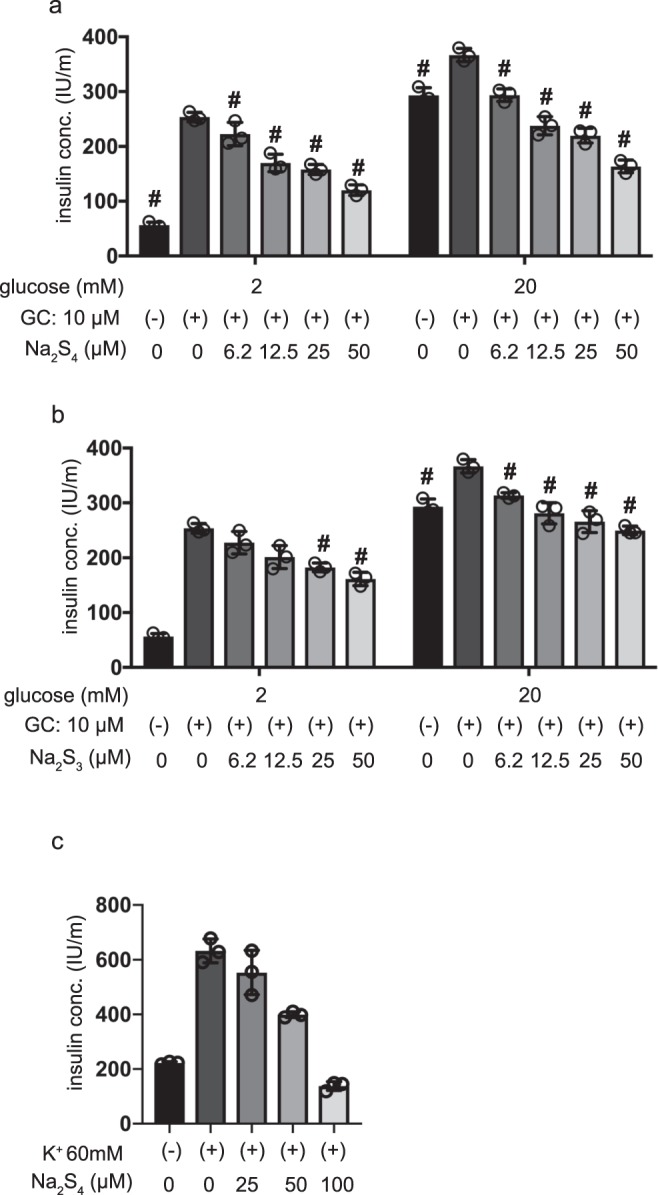
Effects of polysulfide salts on insulin secretion induced by glibenclamide. (**a,b**) Mouse MIN6 cells were exposed to Na_2_S_4_ (**a**) or Na_2_S_3_ (**b**) (0, 6.2, 12.5, 25, or 50 µM) for 4 h without or with glibenclamide (10 µM) under 2 mM or 20 mM glucose conditions (n = 3). (**c**) MIN6 cells were exposed to 60 mM K^+^ with Na_2_S_4_ (n = 3). Insulin secretion was determined as described in Materials and Methods. Data are presented as the mean ± SD (n = 4). Differences between treatments were evaluated by one-way ANOVA followed by Dunnett’s test for multiple comparisons; ^#^*P* < 0.05, as compared with the control (glucose = 2 mM, without glibenclamide or without diazoxide treatment or without high K^+^ treatment).

There is a consensus that the elevation of intracellular ATP/ADP ratio in response to high glucose conditions results in the closure of ATP-sensitive potassium (K_ATP_) channels, and depolarizes the plasma membrane^18^. Glibenclamide, a drug that acts as a channel closer, has been reported to facilitate insulin secretion in pancreatic β-cells even under low-glucose conditions, by binding to and inhibiting the K_ATP_ channel inhibitory regulatory subunit sulfonylurea receptor 1 (SUR1)^14^. The effects of Na_2_S_3_ and Na_2_S_4_ treatment on glibenclamide-induced insulin secretion were examined in MIN6 cells. Here, we found that even upon addition of 2 mM glucose, 10 µM glibenclamide elicited insulin secretion. Such secretion was then suppressed by treatment with more than 6.2 µM Na_2_S_4_ (Fig. 6a). Under 20 mM glucose conditions, Na_2_S_4_ at concentrations exceeding 6.2 µM suppressed glibenclamide-induced insulin secretion (Fig. 6a). As was the case for Na_2_S_4_, high concentrations (>25 µM) of Na_2_S_3_ did suppress glibenclamide-induced insulin secretion in a dose-dependent manner in cells treated with 2 mM glucose conditions (Fig. 6b). In addition, we examined the effect of polysulfide salt on Ca^2+^-triggered insulin secretion by stimulation with 60 mM K^+^. Stimulation with 60 mM K^+^ induced insulin secretion. Insulin secretion was suppressed with 50 µM Na_2_S_4_, but not with 25 µM Na_2_S_4_, which suppressed GSIS (Fig. 6c). These results suggest that polysulfide donors affect cellular processes distal to K_ATP_ channels.

### Effects of Na_2_S_4_ on the membrane potential of MIN6 cells

**Figure 7:**
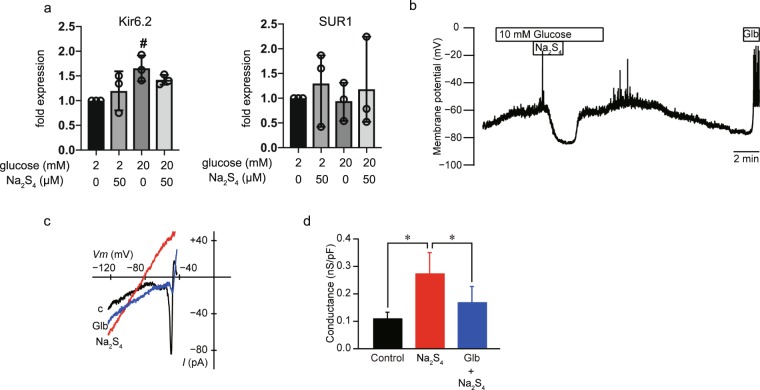
Effects of Na_2_S_4_ on the membrane potential of mouse MIN6 cells. (**a**) Mouse MIN6 cells were exposed to 50 µM Na_2_S with 20 mM glucose and harvested. Then the mRNA levels of Kir6.2, and SUR1 were assayed by *q*RT-PCR. Data are presented as mean ± SD (n = 3). ^#^*P* < 0.05, as compared with the control (Na_2_S_4_ 0 µM). (**b**) Na_2_S_4_ (50 μM) induced hyperpolarization of membrane potential with gramicidin-perforated patch. Glibenclamide (Glb; 100 μM) induced depolarization. These traces are representative of six glucose-responsive cells. (**c**) Representative current–voltage relationships for the whole-cell currents with gramicidin-perforated patch. The current was elicited by a voltage ramp from −123 to −43 mV with a rate of 0.2 V/s. Na_2_S_4_ (50 μM) increased the current in the KRBH buffer contained 10 mM glucose (C; control). Thereafter, the current induced by Na_2_S_4_ decreased by glibenclamide (Glb, 10 μM). (**d**) Summary of the effects of Na_2_S_4_ and glibenclamide on the conductance (n = 9). *P < 0.05 for comparison of the indicated groups.

The expression of Glut2 (*Slc2a2*), Cav1.2 (*Cacna1c*), Kv2.1 (*Kcnb1*), Kv2.2 (*Kcnb2*), Kir6.2 (*Kcnj11*), SUR1 (*Abcc8*), insulin *(Ins1*), and 18 S ribosomal RNA (*Rn18s*) was examined by RT-PCR (Supplementary Fig. 5). Glut2, Cav1.2, Kv2.1, Kv2.2, Kir6.2, SUR1, or insulin expressions were not affected within 4 h of glucose or Na_2_S_4_ treatment (Supplementary Fig. 5). Results from *q*RT-PCR indicated that the expression of K_ATP_ channel interacting intermediates Kir6.2 and SUR1 was not affected by Na_2_S_4_ treatment (Fig. 7a). Kir6.2 expression increased in response to exposure to 20 mM glucose, but was not affected by Na_2_S_4_ treatment (Fig. 7a). These results demonstrate that the expression of molecules which play critical roles in glucose uptake or membrane depolarization was not affected by Na_2_S_4_.

To identify the ion channels affected by Na_2_S_4_, we measured the membrane potential of MIN6 cells using the gramicidin-perforated patch technique (Fig. 7b). Na_2_S_4_ (50 µM) hyperpolarized the membrane potential induced by 10 mM glucose (n = 6). Washing out of Na_2_S_4_ (50 µM) promptly restored the membrane potential depolarization induced by 10 mM glucose, to its original level. Following perfusion with standard 2 mM glucose solution, the firing of action potentials induced by 10 mM glucose disappeared. Glibenclamide (100 µM) addition also induced the firing of action potentials. These results indicated that Na_2_S_4_ activated K_ATP_ channels reversibly.

We measured whole-cell currents with the gramicidin-perforated patch technique and specifically measured K_ATP_ currents in MIN6 cells bathed in a 10 mM glucose solution. Na_2_S_4_ at 50 µM facilitated inwardly rectifying K^+^ currents (n = 9, Fig. 7c). Measured conductance was 0.11 ± 0.22 nS/pF in 10 mM glucose solution (Fig. 7d). Na_2_S_4_ significantly increased the conductance to 0.27 ± 0.08 nS/pF. The addition of 10 µM glibenclamide significantly decreased the conductance to 0.17 ± 0.06 nS/pF (n = 9, Fig. 7d).

**Figure 8:**
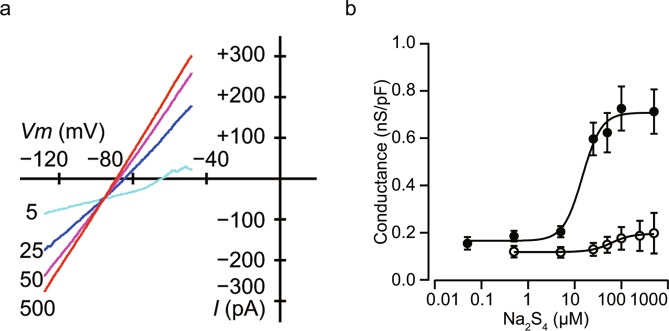
Effect of Na_2_S_4_ on voltage-dependent outward potassium currents in mouse MIN6 cells. (**a**) Current–voltage relationships of whole-cell currents with varying concentrations of Na_2_S_4_ (in 0, 5, 25, 50, or 500 μM). Na_2_S_4_ increased inwardly rectifying potassium currents in a dose-dependent manner (n = 5). (**b**) Semi-logarithmic plot of the conductance between −108 and −68 mV by concentration of Na_2_S_4_ with the standard K^+^ pipette solution included ATP at 0.01 mM (closed circles) and 1 mM (open circles).

Na_2_S_4_ facilitated the inwardly rectifying K^+^ conductance in a dose-dependent manner with an intracellularly standard K^+^ pipette solution that included 0.01 mM ATP, measured with the whole-cell patch-clamp configuration (Fig. 8a). The half maximal effective concentration (EC50) of Na_2_S_4_ was estimated at 16.7 ± 3.1 μM with a Hill coefficient of 5.7 ± 1.7 (n = 5, Fig. 8b). However, the effect of Na_2_S_4_ was suppressed with a standard K^+^ solution containing 1 mM ATP (n = 6, Fig. 8b). Stromatoxin-1 (100 nM, STx), an inhibitor of Kv 2.1 channels, did not decrease the conductance (n = 3, Supplementary Fig. 6). These results together suggest that Na_2_S_4_ affects K_ATP_ channels in MIN6 cells.

## Discussion

In this study, we demonstrated that pharmacological polysulfide salts including Na_2_S_2_, Na_2_S_3_, and Na_2_S_4_ significantly inhibited GSIS in mouse and rat pancreatic β-cell-derived MIN6 and INS-1 cell lines, and that the effect is dependent on activation of the K_ATP_ channel. Moreover, a mixture of Na_2_S and diethylamine NONOate (DEA/NO), which generates polysulfides in cells, also had comparative suppressive effects on GSIS.

Polysulfides, a mixture of substances with varying numbers of sulfurs (H_2_S_n_) is endogenously generated from H_2_S in the presence of oxygen^6,7,19,20^. Polysulfides contain sulfane sulfur, which is maintained in various proteins as a potential intracellular H_2_S store, which releases H_2_S under reduced conditions. It has also been reported that polysulfides are enzymatically biosynthesized by reacting with cysteine^19^. A mixture of Na_2_S (a sodium salt of sulfide) and diethylamine NONOate (DEA/NO, a NO donor), has been reported to generate H_2_S_2_ and H_2_S_3_^7,21,22^. In fact, we demonstrated here that Na_2_S and DEA/NO efficiently inhibited GSIS in MIN6 cells.

Polysulfides, and not H_2_S, have previously been reported to be entities that bind within sulfhydrate or sulfurate proteins^19,20,23^. Polysulfides regulate the function of proteins by modulating cysteine residues in target protein to form -SSH moieties, thereby causing structural change of the protein^1^. In this study, we demonstrated that in fact polysulfides suppressed GSIS far more efficiently than Na_2_S. Na_2_S at a concentration of 50 µM inhibited GSIS but the potency of the inhibition was reduced compared to polysulfides. Notably the potency of the inhibition after treatment with 1.5 µM Na_2_S_4_ was stronger than 50 µM Na_2_S. In fact, the IC_50_ of Na_2_S_4_ was calculated to be 9.5 µM. However, Na_2_S in the presence of an NO donor results in an inhibitory effect similar to the use of polysulfides^4^. Another interesting finding is that the suppressive effect of a polysulfide is related to the number of disulfide bonds present. The suppressive effect of Na_2_S_4_ is stronger than that of Na_2_S_3_ and Na_2_S_4_. In addition, TCEP treatment counteracted the suppressive effect observed in response to polysulfides^24^.

These factors suggest that polysulfide acts as an intermediate species during H_2_S signaling. The present study demonstrates that polysulfides are more potent GSIS suppressors than H_2_S. Polysulfides are known to promote protein sulfhydration more efficiently than H_2_S^25^.

It has also been reported that H_2_S acts as a regulator of O_2_ consumption of mitochondria and intracellular O_2_ metabolism in mammalian cells. In HeLa cells, 100 µM H_2_S was shown to significantly suppress oxygen consumption and affect intracellular ATP concentration^26^. Contrary to this, our experimental results indicate that even 50 µM Na_2_S_4_ failed to significantly influence oxygen consumption or intracellular ATP concentration in MIN6 cells.

Treatment with 50 µM Na_2_S_4_ did not affect the expression of GLUT2, Cav1.2, Kir6.2, or SUR1, nor did it affect cell growth and death. ATP content, OCR, or ECAR, a surrogate marker of glycolysis, was not significantly affected by Na_2_S_4_. The evidence strongly suggests that glucose intake was not affected by polysulfides. In addition, polysulfide treatment did not affect glibenclamide-induced insulin secretion. Taken together, the evidence prompted us to focus on K_ATP_ channels. The K_ATP_ channel is a potassium channel composed of Kir6.x: Kir6.1 and Kir6.2 (a K^+^ channel member belonging to the ‘inward rectifier’ subclass) and sulfonylurea receptor (SUR; SUR1, SUR2A, and SUR2B) subunits^27^. It is sensitive to adenosine triphosphate (ATP), and various subtypes of K_ATP_ channel are expressed in a wide range of tissues^28,29^. In pancreatic β cells, including MIN6 cells and INS-1 cells, K_ATP_ channels are comprised of SUR1 and Kir6.2^28^. Mutations of SUR1 or Kir6.2 genes are known to cause familial hypoglycemia associated with unregulated insulin secretion^14,15,30^. It is reported that H_2_S stimulates K_ATP_ channels in vascular smooth muscle cells and that H_2_S also functions as an endogenous opener of K_ATP_ channels in INS-1E cells. A series of studies has indicated that H_2_S stimulates a variety of ion channels such as TRPA1, TRPV1, T-type Ca^2+^ channels, and K_ATP_ channels^2,3,7,31–33^. The evidence strongly suggests therefore that polysulfides would also affects these ion channels.

In line with this logic, we demonstrated the polysulfides activate K_ATP_ channels in MIN6 cells derived from mouse insulinoma. First, Na_2_S_4_ reversibly hyperpolarized the membrane potential after induction by 10 mM glucose. Second, Na_2_S_4_ facilitated conductance of inwardly rectifying K^+^ currents in a dose-dependent manner upon addition of a standard K^+^ pipette solution that included ATP. Another series of structural studies indicated that Kir6.x subunits sense changes in intracellular ATP concentration^34,35^. In this study, we demonstrated that Na_2_S_4_ facilitated the inwardly rectifying K^+^ conductance in a dose-dependent manner with an intracellularly standard K^+^ pipette solution with 0.01 mM ATP. However, the effect of Na_2_S_4_ was suppressed with a K^+^ pipette solution comprising 1 mM ATP. Conversely, we also demonstrated that 10 µM of glibenclamide significantly decreased inwardly rectifying K^+^ currents elicited by polysulfides. Taking into account existing evidence that the direct target of glibenclamide is SUR, it is reasonable to suggest that polysulfides affect Kir6.2, SUR1, or the interaction between them.

H_2_S is sequentially oxidized to form polysulfides with a varying number of sulfur atoms, until the number of sulfur atoms reaches eight. At that point, sulfur molecules cyclize and separate from polysulfides. Polysulfides also sulfurate cysteine residues in TRPA1 channels to modify their activity^7,31^. Polysulfides sulfurate Cys422 and Cys622 in TRPA1 channels to activate them, and it is reported that vascular K_ATP_ channels are inhibited during oxidative stress caused by S-glutathionylation; the Kir6.1 subunit specifically has been reported to be responsible for oxidant sensitivity in these channels^2,36^. Systematic mutational analysis revealed three cysteine residues in the N terminal and transmembrane domains of Kir6.1: namely Cys43, Cys120, and Cys176. Among them, Cys176 contributed to >80% of the oxidant sensitivity of the protein^36^. In contrast to Cys176, Cys43 exhibited only a modest contribution to S-glutathionylation, and Cys120 was modulated by extracellular oxidants but not by intracellular glutathione disulfide^36^. In Kir6.2 channels, Cys166 (corresponding to Cys176 in Kir6.1) is suggested to be involved in intrinsic channel gating. As in the case of Kir6.1, several cysteine residues are well conserved between species (Supplementary Fig. 8). In addition to Kir6.x, SUR1 also have well-conserved cysteine residues (Supplementary Fig. 7). Notably, the inhibitory effects of polysulfides on GSIS were demonstrated to be reversible in this study. In addition, Na_2_S_4_ activated K_ATP_ channels in MIN6 cells reversibly. Together, the evidence presented here suggests that polysulfides modulate reactive cysteine residues.

There is a limitation of this study. Because we focused on the mechanism of the suppression of insulin secretion by pharmacological polysulfides *in vitro*, we did not perform *in vivo* experiments. *In vivo* experiments using mice may warrant the impact of polysulfides on systemic insulin secretion and glucose metabolism.

## Materials and Methods

### Cell culture

Mouse insulinoma MIN6 cell lines were cultured in Dulbecco’s modified Eagle’s medium (DMEM) (Gibco, Grand Island, NY, USA) containing 450 mg/dl glucose. Rat INS-1 cells were cultured in RPMI1640 (Sigma-Aldrich, St Louis, MO, USA) supplemented with 10% fetal bovine serum (FBS), 50 μM β-mercaptoethanol, 100 U/ml penicillin, and 0.1 mg/ml streptomycin. Culture conditions used replicated those reported in the literature for these cells^37,38^.

### Reagents

Details of reagents used in this study are described in Table S1.

### Isolation of mouse pancreatic islets

Male C57BL/6JJcl mice (8–10 weeks old, n = 8) were sacrificed by cervical dislocation in accordance with protocols approved by the Animal Experimentation Committee, Kansai Medical University (#19–088). Pancreatic islets were isolated from the pancreas by enzymatic digestion of the tissue, using a slight modification to a protocol described by Lacy *et al*.^39^. The pancreas was removed and digested with collagenase (Type IV, 195 U/ml; Worthington Biochemical, Lakewood, NJ, USA) in a solution containing 2 mM glucose and trypsin inhibitor (0.01%; Sigma-Aldrich, St Louis, MO, USA) at 37 °C for 30 min with vigorous shaking. The pancreatic tissue was triturated with a pipette and washed two times with an enzyme-free solution. Islets were selected with a glass micropipette under a stereomicroscope. Batches of ten islets were used to measure insulin concentration.

### Measurement of insulin concentration

Insulin concentration in the culture medium of MIN6, INS-1 cells, and pancreatic β cells/ islets was assayed using a Mouse/Rat Insulin H-type™ enzyme-linked immunosorbent assay kit (Shibayagi Co. Ltd., Shibukawa, Japan), following the manufacturer’s instructions^40^. Detailed protocols are available in the provided supplementary information and at protocols.io (10.17504/protocols.io.v63e9gn).

### Cell viability assay

Cell viability was assessed using the CellTiter 96™ AQueous One Solution Cell Proliferation Assay (Promega, Madison, WI, USA)^41,42^. Cell viability was determined by comparing the absorbance values of the treated cells with those of the control cells (MIN6 cells at 24 h of incubation) defined as 100%. All experiments were performed in triplicate or quadruplicate. Detailed protocols are available in the supplementary information and at protocols.io (10.17504/protocols.io.v64e9gw).

### ATP assay

Intracellular ATP content was evaluated using the CellTiter-Glo™ luminescent cell viability assay kit (Promega)^42^. Assays were performed in triplicate and repeated at least twice. Detailed protocols are available in the supplementary information and at protocols.io (10.17504/protocols.io.v7ke9kw).

### Measurement of cellular oxygen consumption and extracellular acidification

The cellular oxygen consumption rate (OCR) and extracellular acidification rate (ECAR) were measured using an XFp Extracellular Flux Analyzer™ (Agilent Technologies, Santa Clara, CA)^41,43^. MIN6 cells were seeded at a density of 1 × 10^4^ cells/well on an XFp Cell Culture microplate. The XF Cell Mito Stress Test™ was performed in glucose-containing XF base medium, following the manufacturer’s protocol with 1 µM oligomycin, 1 µM FCCP, 0.5 µM rotenone, and 0.5 µM antimycin A. Detailed protocols are available at Supplementary information and protocols.io (10.17504/protocols.io.v92e98e).

### Quantitative reverse transcriptase-PCR (*q*RT-PCR) analysis

Total RNA was isolated using RNeasy™ Mini Kit (Qiagen, Valencia, CA, USA). First-strand cDNA synthesis and real-time PCR were performed as described^41,43^. PCR primers sequences were demonstrated as Table S2 primer sequences for *Actb* (actin, beta; β-actin):, *Abcc8* (ATP binding cassette subfamily C member 8; SUR1):, *Abcc9* (ATP binding cassette subfamily C member 9; SUR2), *Kcnj11* (potassium inwardly rectifying channel, subfamily J member 11; Kir6.2), *Kcnj8* (potassium inwardly rectifying channel, subfamily J, member 8; Kir6.1), *Slc2a2* (solute carrier family 2 (facilitated glucose transporter), member 2; Glut2), and *Cacna1c* (calcium channel, voltage-dependent, L type, alpha 1 C subunit; Cav1.2). Detailed protocols are available at Supplementary information and protocols.io (10.17504/protocols.io.v7ne9me).

### Electrophysiological studies

MIN6 cells were incubated in an extracellular bath solution containing 2 mM glucose for 30 min at 37 °C before patch-clamp experiments^44–46^. Membrane potential measurements and whole-cell current recordings were performed using the EPC 800 patch-clamp amplifier (HEKA Elektronik Inc. Holliston, MA, USA). Experiments were conducted at 23–30 °C. Detailed protocols are available at Supplementary information and protocols.io (10.17504/protocols.io.v68e9hw).

### Statistical analysis

Data are presented as means ± SD. Differences between groups were evaluated with one-way analysis of variance (ANOVA) and two-way ANOVA followed by Dunnett’s test for multiple comparisons. Statistical analyses were performed with Prism8™ (GraphPad Software, Inc. La Jolla, CA). Statistical significance was defined by *P*-values < 0.05.

## Supplementary information

Supplementary Information

## Data Availability

The datasets analyzed in this study are available in the Supplementary Information and the corresponding author upon reasonable request.
