# Supplementary material for: Pharmacological polysulfide suppresses glucose-stimulated insulin secretion in an ATP-sensitive potassium channel-dependent manner
Source: Sci Rep. 2019 Dec 18;9:19377. doi: 10.1038/s41598-019-55848-7 (PMC6920347; doi:10.1038/s41598-019-55848-7)
Supplement: Supplementary file 1 — Supplementary Information [file 41598_2019_55848_MOESM1_ESM.docx]

**Supplementary Information**

Pharmacological polysulfide suppresses glucose-stimulated insulin secretion in an ATP-sensitive potassium channel-dependent manner

Tomohiro Shoji^1, 2^, Mikio Hayashi^3^, Chisato Sumi^1,2^, Munenori Kusunoki^1,2^, Takeo Uba^1,2^, Yoshiyuki Matsuo ^2^, Hideo Kimura^4,5^, Kiichi Hirota^2^

^1^ Department of Anesthesiology, Kansai Medical University, Hirakata, Japan

^2^ Department of Human Stress Response Science, Institute of Biomedical Science, Kansai Medical University, Hirakata, Japan

^3^ Department of Cell Physiology, Institute of Biomedical Science, Kansai Medical University, Hirakata, Japan

^4^ Department of Pharmacology, Faculty of Pharmaceutical Science, Sanyo-Onoda City University, Sanyo-Onoda, Japan

^5^ Department of Molecular Pharmacology, National Institute of Neuroscience, National Center of Neurology and Psychiatry, Kodaira, Japan

*Corresponding author: Kiichi Hirota: hif1@mac.com

**Supplementary Informations**

**Table S1 Key Resources Table**

| Reagents | Source | Identifier |
| --- | --- | --- |
| Sodium tetrasulfide (Na_2_S_4_) | Dojindo | 12034-39-8 |
| Sodium trisulfide (Na_2_S_3_) | Dojindo | 37488-76-9 |
| Sodium disulfide (Na_2_S_2_) | Dojindo | 22868-13-9 |
| Sodium sulfide (Na_2_S) | Dojindo | 1313-82-2 |
| Apo-ONE™ Homogeneous Caspase-3/7 Assay Kit | Promega | G7792 |
| albumin from bovine serum (BSA), fatty acid free | Wako | 017-15141 |
| 45w/v% D(+)-Glucose | Wako | 079-05511 |
| CellTiter-Glo™ luminescent cell viability assay kit | Promega | G7570 |
| Dimetyl Sulfoxide | Nacalai Tesque | 13406-55 |
| Dulbecco’s modified Eagle’s medium | Thermo Fisher Scientific | 11965-092 |
| ethylenediamine tetraacetic acid (EDTA) | Dojindo | H001 |
| fetal bovine serum | GE Healthcare | SH30910 |
| glibenclamide | Wako | 078-03881 |
| gramicidin D | Sigma-Aldrich | G5002 |
| HEPES | Nacalai Tesque | 17557-94 |
| K_2_ATP | Sigma-Aldrich | A8937 |
| KCl | Wako | 163-03545 |
| KH_2_PO_4_ | Wako | 7778-77-0 |
| KOH | Nacalai Tesque | 28616-45 |
| L-glutamine | Nacalai Tesque | 16948-04 |
| Lidocaine | TERUMO | 2129409G1036 |
| MgSO_4_ | Wako | 132-00435 |
| NaCl | Wako | 191-01665 |
| NaH_2_PO_4_・2H_2_O | Wako | 192-02815 |
| NaHCO_3_ | Nacalai Tesque | 312-13 |
| NaOH | Sigma-Aldrich | 28-3010-5 |
| penicillin-streptomycin | Nacalai Tesque | 09367-34 |
| potassium aspartate | Sigma-Aldrich | A6558 |
| RPMI medium | Thermo Fisher Scientific | 11875-093 |
| sodium pyruvate | Nacalai Tesque | 06977-34 |
| stromatoxin-1 | Alomone Labs | STS-350 |
| β-Mercaptoethanol | Wako | 131-14572 |
| Diethylamine NONOate | Cayman | 372965-00-9 |
| GYY4137 | Dojindo | 106740-09-4 |
| Immobilized TCEP Disulfide Reducing Gel | Thermo Fisher Scientific | 77712 |
| Sodium sulfide (Na_2_S) | Dojindo | 1313-82-2 |
| Bond-Breaker® Tris[2-Carboxyethyl]phosphine Neutral Solution (TCEP) | Pierce™ | 77720 |

**Table S2 | PCR primers**

Actb (β-actin)

FW:5’-TCCTTCTTGGGTATGGAATCCT-3’

RV:5’-GTCTTTACGGATGTCAACGTCAC-3’

Kcnj8 (Kir6.1)

FW:5’-CGCTGTCTGTGTGACCAATG-3’

RV:5’-CAAAACCGTGATGGCCAGAG-3’

Kcnj11 (Kir6.2)

FW:5’-GACATCCCCATGGAGAATGG-3’

RV:5’-TCGATGACGTGGTAGATGATGAG-3’

Abcc8 (SUR1)

FW:5’-GGAGTGGACAGGACTGAAGG-3’

RV:5’-AGTCAAGGCGGAGACACAGA-3’

Abcc9 (SUR2)

FW:5’-ACCGGAGTGCAATCAAAACC-3’

RV:5’-AAACCACTGCCCCATGAGAA-3’

Kcnb1 (Kv2.1)

FW:5’-AGAAACACACAGCAATAGCGT-3’

RV:5’-GTACTCCCGTGGAGACTCTTG-3’

Kcnb2 (Kv2.2)

FW:5’-ACTGTAACACTCACGAGAGTCT-3’

RV:5’-CTCCAGGATGTCGGTCGAAG-3’

Ins1 (Insulin)

FW:5’-CCCTTAGTGACCAGCTATAATCAGAGA-3’

RV:5’-ACCACAAAGATGCTGTTTGACAA-3’

Slc2a2 (Glut2)

FW:5’-TCAGAAGACAAGATCACCGGA-3’

RV:5’-GCTGGTGTGACTGTAAGTGGG-3’

Cacna1c (Cav1.2)

FW:5’-GAAGGCACTGAGAGCGTTCC-3’

RV:5’-GCTCCAGGCCGATAATAGCAT-3’

Supplementary Figure 1


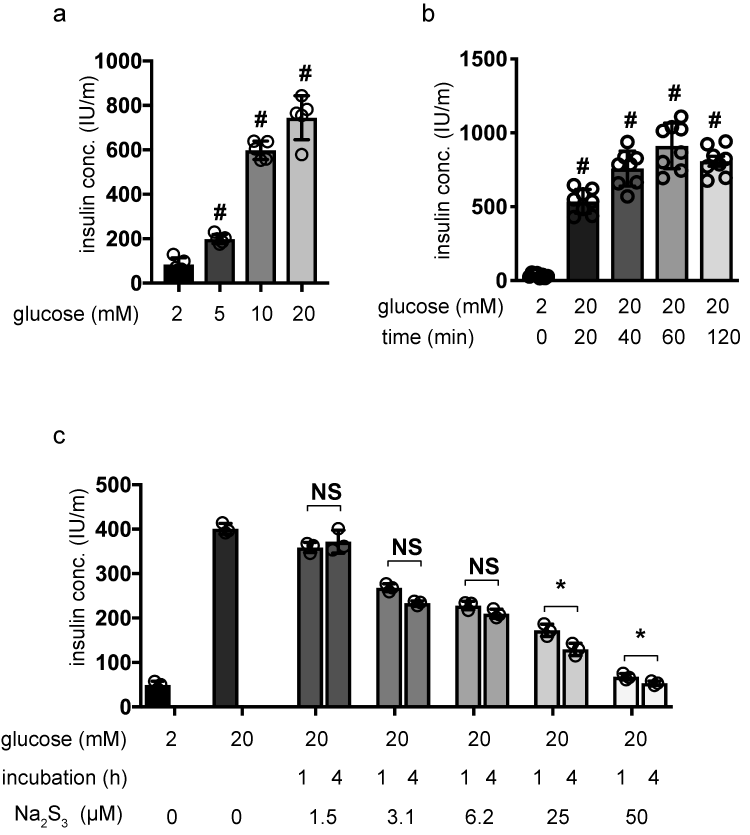


Supplementary Figure 1 | Profile of glucose-stimulated insulin secretion in MIN6 cells

(a) MIN6 cells were stimulated with the glucose concentrations shown for 1 h. (b) MIN6 cells were incubated with 20 mM glucose for the times shown prior to calculation of insulin secretion. (c) MIN6 cells were exposed to Na_2_S_3_ (0, 1.5, 3.12, 6.2,12.5, 25 and 50 µM) for 1 hour or 4 hours and then insulin secretion was determined under 20 mM glucose conditions. Insulin secretion was determined as described in Materials and Methods. Data are presented as mean ± SD (n = 5). Differences between treatments were evaluated by one-way ANOVA followed by Dunnett’s test for multiple comparisons; #*P* < 0.05, as compared with the control ((a): glucose = 2 mM; (b): time = 0 min) or **P* < 0.05 for comparison of the indicated groups

Supplementary Figure 2


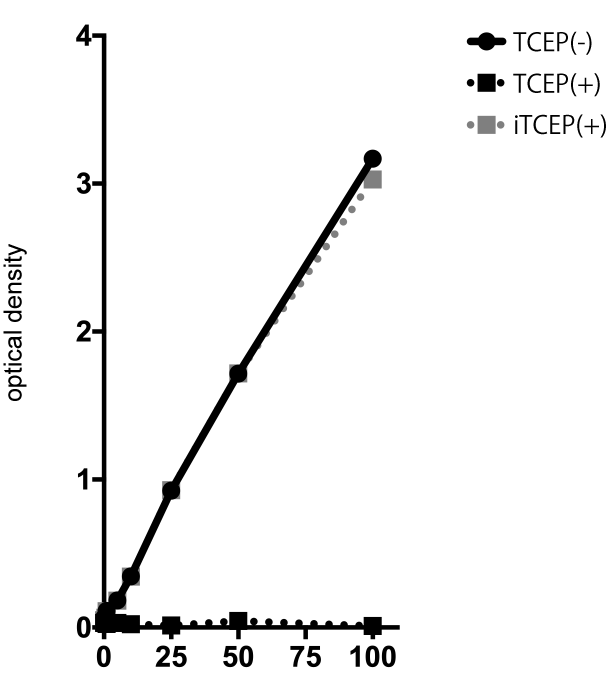


Supplementary Figure 2 | Effect of TCEP on insuline measurement

Define concentration of insulin was exposed to 250 µM tris (2-carboxyethyl) phosphine hydrochloride (TCEP) or iTCEP for 1 hour and then insulin secretion was determined.

Supplementary Figure 3


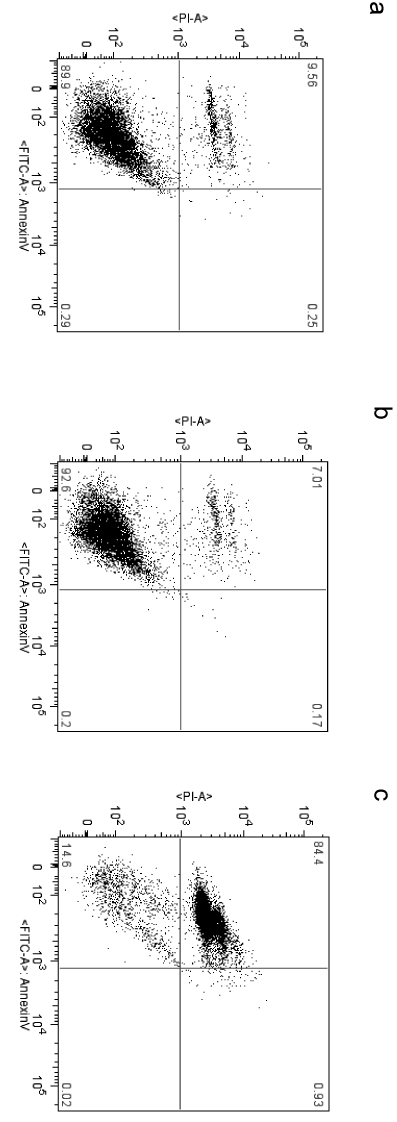


Supplementary Figure 3 | Effect of Na_2_S_4_ on cell death

MIN6 cells were exposed to 0 (a) or 100 µM (b) Na_2_S_4_ or 10 mM lidocaine (c) for 4 h. Cells were harvested, and percentages of cell death were measured by flow cytometry. The ratio of PI-positive and/or annexin V-positive cells [(Q1 + Q2 + Q4)/(Q1 + Q2 + Q3 + Q4)] was used to calculate the percentage of dead cells.

Supplementary Figure 4


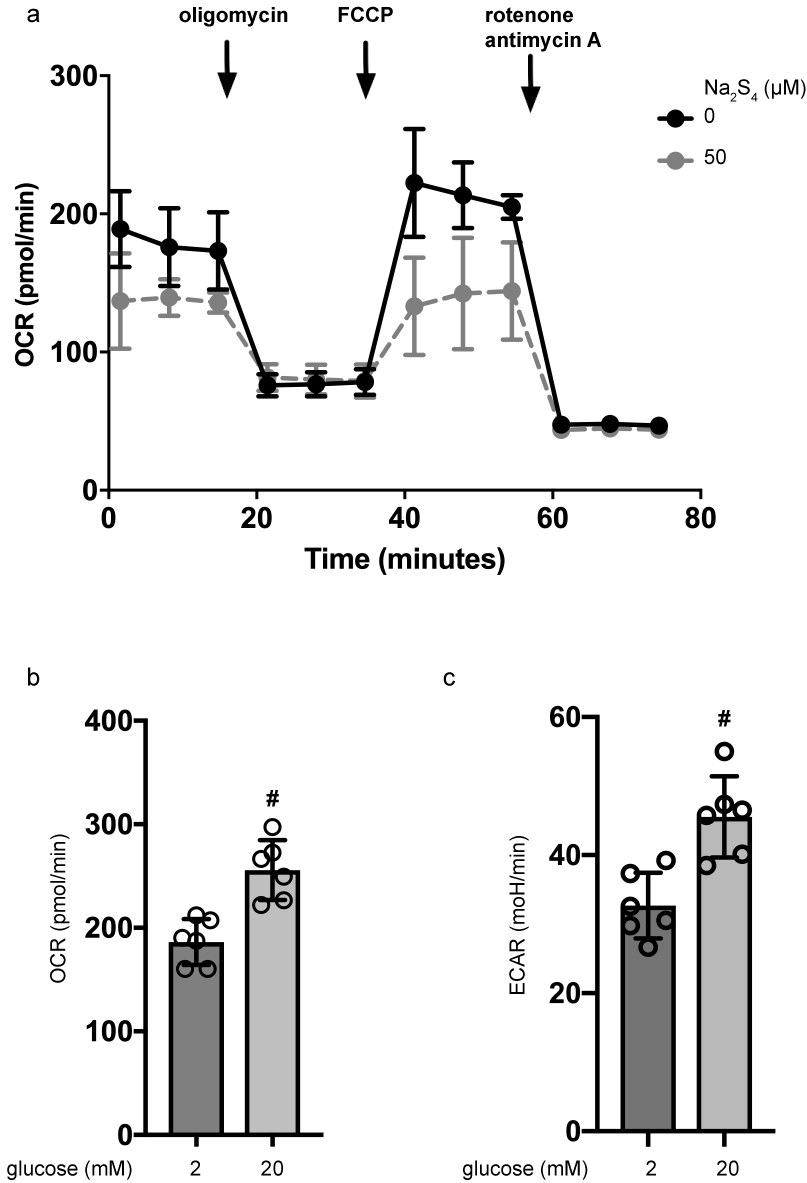


Supplementary Figure 4 | Effects of polysulfide salts on oxygen metabolism in MIN6 cells

(a) Cell Mito Stress test profile of the key parameters of mitochondrial oxygen consumption rate (OCR). 1 µM oligomycin, 1 µM FCCP, 0.5 µM rotenone and 0.5 µM antimycin A (b) Basal OCR was calculated. (c) Basal ECAR was calculated. Data presented are expressed as means ± standard deviations (SD). Differences between results were evaluated by Student *t*-test #*p* < 0.05 compared to the control cell population.


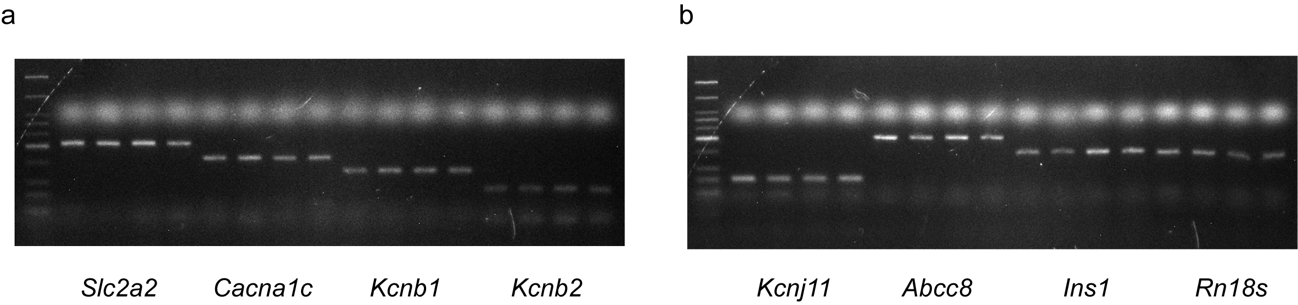
Supplementary Figure 5

Supplementary Figure 5 | RT-PCR of Glut2 (*Slc2a2*), Cav1.2(*Cacna1c*), Kv2.1(*Kcnb1*), Kv2.2(*Kcnb2*), Kir6.2 (*Kcnj11*), SUR1 (*Abcc8*), insulin (*Ins1*), and 18S ribosomal RNA(*Rn18s*) mRNA

Mouse MIN6 cells were exposed to 50 µM Na_2_S_4_ under 2 or 20 mM glucose conditions and harvested. Then the mRNA levels of Glut2 (*Slc2a2*), Cav1.2(*Cacna1c*), Kv2.1(*Kcnb1*), Kv2.2(*Kcnb2*), Kir6.2 (*Kcnj11*), SUR1 (*Abcc8*), insulin (*Ins1*), and 18S ribosomal RNA(*Rn18s*) mRNA were assayed by RT-PCR.

Supplementary Figure 6


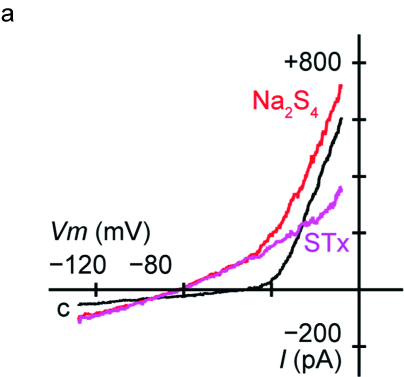


Supplementary Figure 6 | Effect of Stromatoxin-1 on the K^+^ currents

Stromatoxin-1 (100 nM, STx), an inhibitor of Kv 2.1 channels, did not decrease the conductance between −123 to −63 mV but blocked voltage-dependent conductance (n = 3).

Supplementary Figure 7


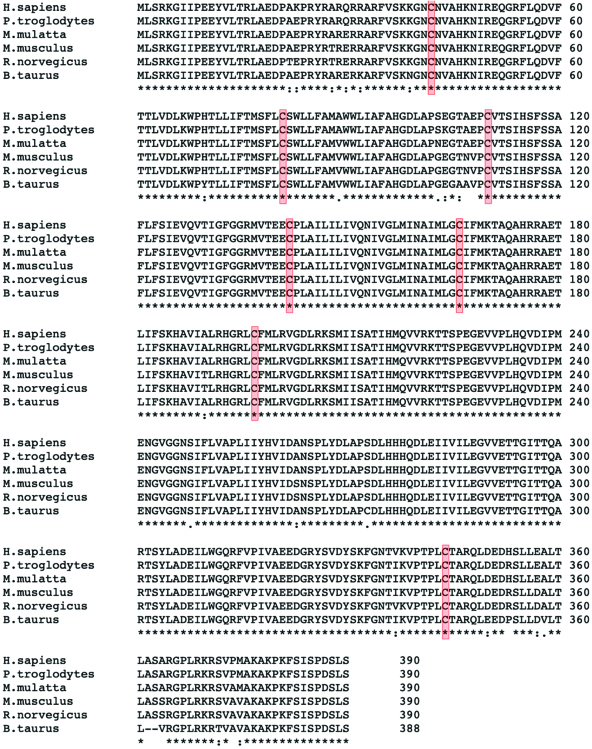


Supplementary Figure 7 | Multiple sequence alignments of mammalian SUR1 proteins

Amino acid sequences of SUR1 from 6 mammalian species (Homo sapiens, NP_001274103; Pan troglodytes, XP_016776002; Macaca mulatta, XP_014970248; Mus musculus, NP_035640; Rattus norvegicus, NP_037171; Bos taurus, NP_001192539) were aligned using Clustal Omega (Sievers F. et al. 2011, Mol Syst Biol. 7:539. DOI: 10.1038/msb.2011.75).

Supplementary Figure 8


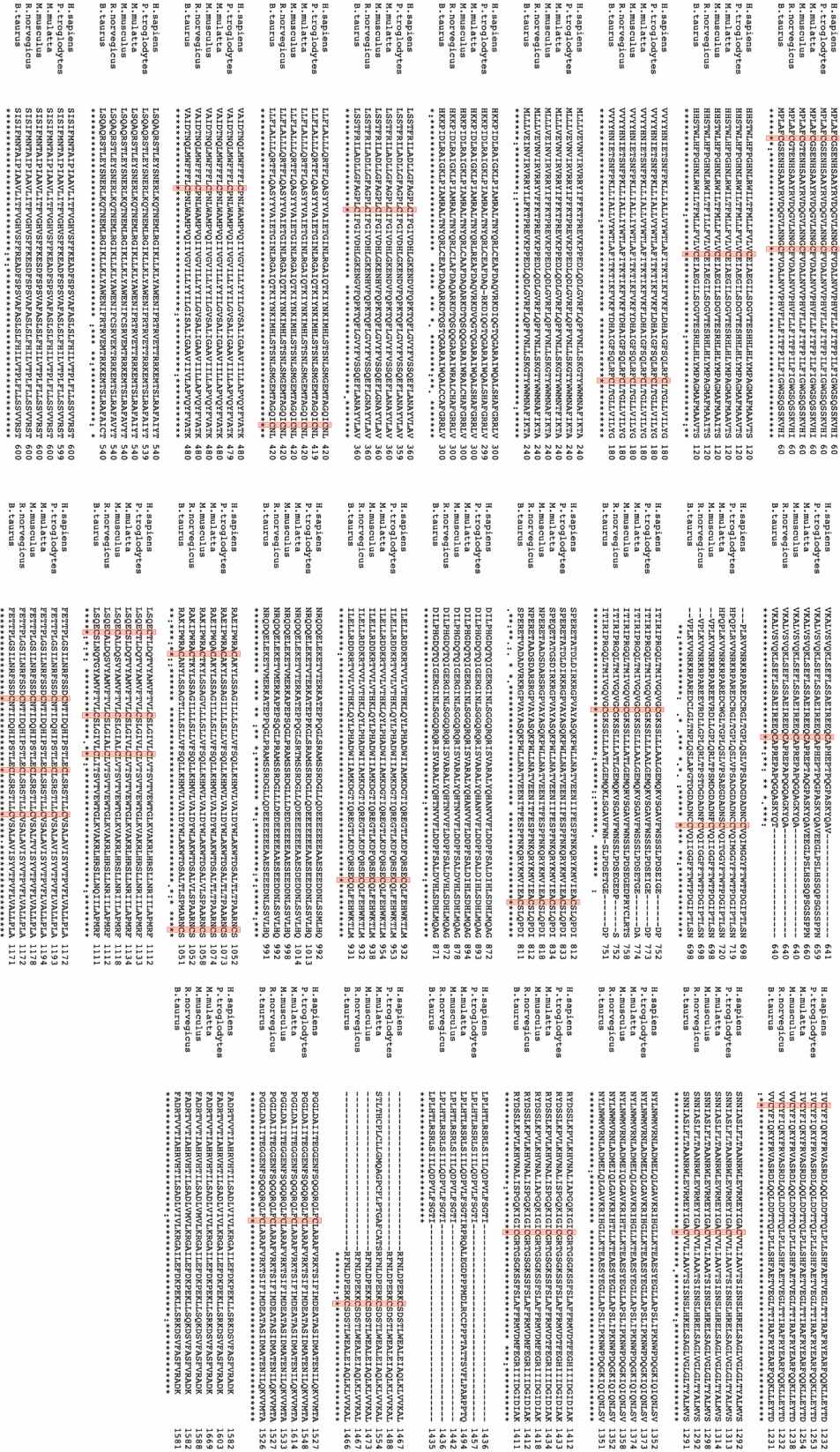


Supplementary Figure 8 | Multiple sequence alignments of mammalian KIR6.2 proteins.

Amino acid sequences of KIR6.2 from 6 mammalian species (Homo sampiens, NP_000516; Pan troglodytes, XP_521849; Macaca mulatta, NP_001248544; Mus musculus, NP_034732; Rattus norvegicus, NP_112648; Bos taurus: NP_001075067) were aligned using Clustal Omega (Sievers F. et al. 2011, Mol Syst Biol. 7:539. DOI: 10.1038/msb.2011.75).
